# Arsenic and Cadmium in Food-chain in Bangladesh—An Exploratory Study

**DOI:** 10.3329/jhpn.v28i6.6606

**Published:** 2010-12

**Authors:** Shafiqul Islam Khan, A.K. Mottashir Ahmed, Mohammad Yunus, Mahfuzar Rahman, Samar Kumar Hore, Marie Vahter, M.A. Wahed

**Affiliations:** ^1^ Laboratory Sciences Division; ^2^ Public Health Sciences Division, ICDDR,B, Mohakhali, Dhaka 1212, Bangladesh; ^3^ Institute of Environmental Medicine, Karolinska Institutet, Stockholm, Sweden; ^4^ Department of Epidemiology, Mailman School of Public Health, Columbia University, NY, USA; ^5^ Health and Nutrition Wing, Hodavasi Chawdhury & Company, Dhaka, Bangladesh

**Keywords:** Arsenic, Arsenic contamination, Cadmium, Cooking process, Food, Bangladesh

## Abstract

Arsenic contamination of tubewell water is a major public-health problem in Bangladesh. In the recent years, the use of shallow and deep tubewell water for irrigation and the use of excess amount of cheap fertilizers and pesticides containing cadmium pose a serious threat of contamination of arsenic and cadmium in food. In an exploratory study, arsenic and cadmium were measured in foods from Matlab, a rural area in Bangladesh, that is extensively affected by arsenic and the economy is agriculture-based. Raw and cooked food samples were collected from village homes (households, n=13) and analyzed to quantify concentrations of arsenic and cadmium using atomic absorption spectrophotometry. Washing rice with water before cooking reduced the concentration of arsenic in raw rice by 13–15%. Rice, when cooked with excess water discarded, showed a significant decrease in arsenic concentration compared to that cooked without discarding the water (p<0.001). In contrast, concentration of cadmium did not decrease in cooked rice after discarding water. Cooked rice with discarded water had significantly lower concentration of arsenic compared to raw rice (p=0.002). Raw rice had higher concentration of arsenic compared to raw vegetables (p<0.001); however, no such difference was found for cadmium. Compared to raw vegetables (e.g. arum), concentration of arsenic increased significantly (p=0.024) when cooked with arsenic-contaminated water. Thus, the practice of discarding excess water while cooking rice reduces the concentration of arsenic but not of cadmium in cooked rice. However, water generally not discarded when cooking vegetables to avoid loss of micronutrients consequently retains arsenic. The results suggest that arsenic and cadmium have entered the food-chain of Bangladesh, and the cooking practices influence the concentration of arsenic but not of cadmium in cooked food.

## INTRODUCTION

Arsenic contamination of groundwater is a major public-health concern in Bangladesh and elsewhere (1–3). Chronic toxicity of arsenic in humans from arsenic-contaminated drinking-water occurs in 61 of 64 districts in Bangladesh, affecting millions of people (2). The maximum permissible level of arsenic in drinking-water recommended by the World Health Organization (WHO) is 10 μg/L and, in Bangladesh, it has been adjusted to 50 μg/L by the local authorities (3). According to the Joint Food and Agriculture Organization/WHO Expert Committee on Food Additives (JECFA), the previous provisional tolerable weekly intake (PTWI) for inorganic arsenic was 15 μg/kg body-weight (equivalent to 2.1 μg/kg body-weight per day or ca. 130 μg per day for a subject of 60 kg body-weight). However, since the previous PTWI is no longer appropriate, the Committee has withdrawn it.

In its 72nd meeting, held in Rome, on 16–25 February 2010 (Summary and conclusions, issued on 16 March 2010), the JECFA determined a lower limit for inorganic arsenic on the benchmark dose for a 0.5% increased incidence of lung cancer (BMDL_0.5_) of 3.0 μg/kg body-weight per day, i.e. 2–7 μg/kg body-weight per day, based on the range of estimated total dietary exposure (4).

Where shallow groundwater is contaminated, it is likely that arsenic is present in bioavailable forms in soil and in irrigation water (5). During the 1960s-1980s, shallow tubewells were installed in Bangladesh to provide ‘safe water’ to prevent morbidity and mortality due to gastrointestinal diseases caused by contaminated surface water. Water from these tubewells was not tested for arsenic or other toxic element contamination.

Results of studies in countries where the population has had long-term exposure to arsenic in groundwater indicate that 1 in 10 people who drinks water containing 500 μg/L of arsenic may ultimately die due to arsenic-induced cancer of lung, bladder, skin and cardiovascular diseases (1, 3). A recent survey showed that arsenic-related diseases resulted in 9,136 deaths per year and 174,174 disability-adjusted life-years (DALYs) among people who were exposed to arsenic concentrations of above 50 μg/L, and this constituted about 0.3% of the total burden of disease in Bangladesh (6).

In Bangladesh and elsewhere, exposure to arsenic may involve a number of pathways: (a) by ingestion of contaminated drinking-water and food and (b) by inhalation of metal-containing dust. After a massive safe campaign by the United Nations Children's Fund and other donor organizations, the installation of irrigation tubewells—both deep and shallow—largely began during the decade of 1980 (7). With the increased use of groundwater through irrigation-pumps during the 1990s, arsenic contamination of water at different depths of soil surface was observed (1, 7).

Later in the 2000s, the use of water from both shallow and deep tubewells for irrigation of agricultural lands began, particularly during the dry season (8). In all likelihood, the use of arsenic-contaminated water for irrigation may have contributed substantially to the spread of arsenic in the top soil-to crop-to food (9, 10). Simultaneously, the use of huge amounts of chemical fertilizers and pesticides also increased dramatically as the crops of high-yielding variety are less resistant to pests. We hypothesized that the excess use of fertilizers and pesticides might lead to accumulation of toxic elements, such as cadmium in soil, that may eventually reach the food-chain. Thus, in this pilot study, we aimed at assessing the concentrations of arsenic and cadmium in rice and vegetables collected from rural Matlab—both in raw and cooked form.

## MATERIALS AND METHODS

### Sampling site

As part of a study published earlier (11), both raw and cooked vegetables and a few traditionally-cooked rice samples were collected from two villages. As described elsewhere (12), the tubewells of those villages showed high concentration of arsenic (81–96% yielded water with more than 50 μg As/L), and the vast majority of farmers used contaminated groundwater for irrigation.

### Sample-collection procedure

A structured questionnaire was employed for each of the above households in the villages for collecting information on the type and quantity of water and foods ingested, and how the foods were being cooked for their consumption. A sample-collection form was used for documenting the address of the household head, type of food, and way of processing (11, 12).

Food items were selected based on usual daily dietary habit and those available around courtyard. About 250 g of rice (*Oryza sativa*) and the edible part of each vegetable, such as amaranth (*Amaranthus viridis*), bitter gourd (*Momordica charantia*), arum (*Colocasia esculenta*) stem, potato (*Solanum tuberosum*), spinach (*Basella alba*), green banana (*Musa* spp.), and eggplant (*Solanum melongena*), were collected from various households (n=13) in polyethylene bags. Parts of rice samples were collected as raw and parts as cooked in accordance with the cooking customs of the population, which was to discard excess water after cooking. Cooked vegetable was also collected. Both tubewell water and surface water were used during cooking. The samples were transferred to the laboratory and washed with deionized water to remove remains of soil. Both raw and cooked samples were frozen at −20 °C until analysis (13).

### Elemental analysis

Deionized water (18.2 megohm-cm) obtained with a glass distillation and E-pure water system (Barnstead, USA) was used for the preparation of reagents and standards. HNO_3_ 69%, HClO_4_ 71%, HCl 36%, NaBH_4_ 98%, KI 99.5%, NaOH 99%, arsenic pentoxide, and cadmium standard solution (1,000 mg/L) of analytical grade were purchased from VWR Int. Ltd., UK.

The arsenic concentration was measured using an atomic absorption spectrophotometer (AA-6800, Shimadzu, Kyoto, Japan) equipped with an auto-sampler (ASC-6100, Shimadzu), using hydride vapour generation (HVG-1, Shimadzu). For cadmium, a graphite furnace (GFA-EX7, Shimadzu) was used, along with the atomic absorption spectrophotometer.

Calibration standards for all the solutions were prepared from the working standard 1,000 μg/L. In the case of arsenic determination, to 10 mL of each solution (sample, recovery, blank, standard) 1 mL of 5M HCl and 1 mL of 20% KI (w/v) were added and heated in water bath at 80°C for 30 minutes to reduce As (V) to As (III) and cooled to room temperature for analysis of arsenic. The detection limit of arsenic for food samples was found to be 0.3 μg/kg in solution. Hydride vapour generation-atomic absorption spectrophotometry (HVG-AAS) showed excellent correlation coefficients between 0.9998 and 0.9996 over the concentration range of 2 μg/L to 15 μg/L of arsenic. However, necessary dilutions were made for samples containing high concentration of arsenic. To validate the precision and accuracy of the method, recovery and duplicate food samples were analyzed (recovery=97%, CV%=±5). For the determination of cadmium, graphite furnace-atomic absorption spectrophotometry (GF-AAS) was used, and the method followed was described earlier (14–16). Standard reference materials—SRM 1643e (trace elements in water) and SRM 1568a (rice-flour) from NIST (USA)—were used for checking the precision and accuracy of the analysis, which were found to be excellent with CV%=±5.

### Methods of cooking

To evaluate the effects of arsenic concentration from cooked rice, we performed in the laboratory two conventional cooking processes of rice in Bangladesh to understand the extent of arsenic removal. The third process was done at the household level in our study area.

Process A: An excessive volume of water was used for cooking rice, and excess water was discarded, which is the tradition of the study households. Before cooking, raw rice is washed with water until water is clear and no longer turbid. The water used for washing and cooking arsenic-contaminated rice was arsenic-free. The rice-washed water, discarded water, and cooked rice were analyzed for arsenic, and in the case of cadmium, raw rice and cooked rice were analyzed.

Process B: An optimum volume of water was used for cooking rice so that no water was needed to be discarded. The rice-washed water and cooked rice were analyzed for arsenic.

Process C: Water generally is not discarded when cooking vegetables as it is perceived that discarding excess water would lead to loss of micronutrients. Cooked vegetables were collected from households as mentioned above and analyzed for arsenic and cadmium.

Before analysis, all the samples were treated as described earlier (15).

### Statistical analysis

Statistical analyses were done using the SigmaStat software (version 3.1) (Systat Software, Inc.). Descriptive frequencies of the study variables were analyzed to assess validity of data, distributions, and assumptions of normality and equal variance. Concentrations of arsenic and cadmium were expressed as mean±standard deviation in raw and cooked food items. Between-groups comparison was done by Student's *t*-test, and for within-groups comparison, paired *t*-test was done. The overall significance level of these tests was set at <0.05.

## RESULTS

### Cooking processes

Cooking process A and B were compared using arsenic-free water in the laboratory. Washing rice until water became clear reduced the concentration of arsenic in raw rice by 13–15%. The conventional cooking process A removed 54% of arsenic in the discarded water (Table 1 and Fig.). The mean concentration of arsenic in raw rice was 240.8 (range 219–253) μg/kg whereas, in cooked rice samples, it was 56.5 (range 18–78) μg/kg.

**Table 1.** Percentage distribution of arsenic in rice and water after different cooking processes

Process*	Rice-washed water	Water discarded	Arsenic retained in cooked rice
A	13	54	33
B	15	Not discarded	85

*Contaminated rice, cooked with arsenic-free water;

Process A: Excess water discarded during cooking.

Process B: An optimum volume of water used for cooking. No water needed to be discarded after cooking

**Fig.**
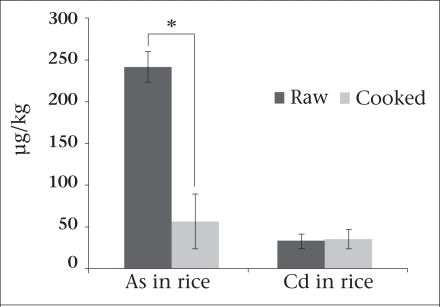
Concentrations of arsenic and cadmium in raw and cooked rice (cooked by process A)

Data are expressed as mean±standard deviation in bars.
*Denotes the significant difference (p=0.002) of arsenic between raw and cooked rice;
As=Arsenic;
Cd=Cadmium

### Arsenic concentration in raw and cooked vegetables

The ranges of arsenic concentrations (μg/kg) detected in raw leafy vegetables (amaranth, spinach, and arum) and non-leafy vegetables (potato, bitter gourd, banana, and eggplant) are given in Table 2. Among the cooked vegetables, the highest concentration of arsenic was found in amaranth (309 μg/kg). Since arsenic-contaminated water was used for cooking vegetables, the concentration of arsenic in cooked vegetables increased and was significantly higher than that in raw vegetables (p=0.024). The increase in arsenic concentration in the cooked spinach, amaranth (leafy part), and arum was about 193%, 539%, and 1470% respectively.

**Table 2.** Concentrations of arsenic in raw and cooked vegetables

Item	No.	Raw (μg/kg)		Cooked (μg/kg)		% of increase after cooking
		Range	Mean±SD	Range	Mean±SD	
Amaranth	8	18–26	22.8±3.7	62–309*	145.6±141	539
Arum	9	6–7	7.03±0.07	49–236*	110.3±85	1,470
Spinach	8	14–16	15.1±0.6	36–49	44.2±6.3	193
Amaranth stem	4	0.5–3	1.3±1.1	NA	NA	NA
Banana	4	4–7	5.3±1.6	NA	NA	NA
Bitter gourd	4	1–6	3.1±2.6	NA	NA	NA
Eggplant	4	3–9	7.0±3.0	NA	NA	NA
Potato	4	4–6	5.4±0.4	NA	NA	NA

*Food cooked using arsenic-contaminated tubewell water. The tubewells of those villages showed high arsenic concentration (81–96% yielded water with more than 50 μg As/L);

NA=Not analyzed;

SD=Standard deviation

The mean concentration of arsenic in raw rice was significantly higher than that in raw vegetables (p<0.001).

### Cadmium in foodstuff

Assessment of cadmium in rice and vegetables (Table 3) showed that the mean cadmium concentration levels were similar in the raw rice (33.1 μg/kg) and raw vegetables (27 μg/kg) (p=0.6). Rice cooked by process A (discarding excess water) did not reduce the concentration of cadmium in the cooked rice (Fig.).

**Table 3.** Concentrations of cadmium in raw andcooked rice and vegetables

Food	Type	Cadmium (μg/kg)
Rice	Raw	33.1±8.5
Rice	Cooked*	34.8±12.2
Amaranth	Raw	33.0±1.0
Bitter gourd	Raw	21.1±0.5
Eggplant	Raw	27.0±1.8

*Cooked by process A

## DISCUSSION

The results showed that the practice of discarding excess water while cooking rice reduced the concentration of arsenic but not of cadmium in the cooked rice. Again, not discarding water when cooking vegetables to avoid loss of micronutrients retained arsenic in cooked vegetables.

In the present study, we found high concentrations of arsenic in rice-grains collected from Matlab, suggesting that rice is being contaminated with arsenic. This might be due to the contaminated water from deep and shallow tubewells used for irrigation. The cooking process also affects the concentration of arsenic in rice. In our study, cooking process A removed 67% of arsenic from the cooked rice during washing with arsenic-free water and by discarding excess water. In process B, we found that 85% of the total arsenic was retained in the cooked rice, thereby significantly increasing the risk of arsenic ingestion through contaminated rice-grains and by the cooking process. The cooking process A, used widely in rural Matlab, is a good practice for reducing contamination of arsenic in cooked rice, provided arsenic-free water is used. Earlier studies have also shown that cooking of rice following the traditional method of the subcontinent, i.e. process A, eliminated up to 54–57% of the total arsenic from cooked rice (17, 18).

The arsenic-contaminated rice may be considered a catastrophic situation in South-East Asia where concentration of arsenic in underground water is high, and rice being the staple food, could be an emerging threat locally and globally since rice is exported from these countries to many western regions/countries, including Europe and the United States (18).

The cooking process C is commonly practised in rural areas in which water is not discarded while cooking vegetables to avoid loss of micronutrients. This cooking process increases the concentration of arsenic in cooked vegetables and, thus, water sources being used for cooking vegetables have a great impact on the concentration of arsenic in food.

We found that raw rice contained higher concentration of arsenic compared to raw vegetables. This might be due to two reasons. First, the vegetables that were collected from households in Matlab were grown in the vicinity of the homes (home-gardening), and pond- or river-water containing low levels of arsenic was used for irrigating vegetables, unlike rice grown in large fields where arsenic-contaminated underground water is used for irrigation. Second, paddy has the enhanced capacity to accumulate arsenic compared to other cereal crops that contain lower arsenic concentration (19). Plants are known to have differential absorption and translocation of arsenicals to its various parts, e.g. roots, stems, and leaves (20, 21), which may explain the higher levels of arsenic in leafy vegetables than in non-leafy vegetables. Metal transporter genes that translocate arsenic into and out of the plants may also play a role in the accumulation of arsenic in different parts of vegetables (22). Moreover, the intake of arsenic and other toxic elements by plants from soil varies from region to region. Some types of soil have a capacity for very strong bonding while others do not (23). The use of water from both shallow and deep tubewells for irrigation of agricultural lands, particularly during the dry period (November-March) for production of high-yielding varieties of rice augment the accumulation of excessive level of arsenic (8). Flooding changes the chemistry of soil dramatically and makes arsenic more soluble; thus, recurrent flooding in Bangladesh redistributes and mobilizes arsenic in the surface-layer of soil from highly-exposed zones to low-exposure zones, especially via drainage from contaminated sources (24, 25). Rice grown in flooded fields picks up more arsenic than rice grown in unflooded fields (24). Ponds may also be contaminated with arsenic where arsenic-contaminated tubewells are installed near the ponds. The inflow of drainage from tubewells was found to be the major cause of arsenic contamination in pond-water (25).

Cadmium is a pollutant which has strong negative effects on human and animal health. Chronic exposure to cadmium is associated with kidney damage, bone damage, cancer, low birthweight, spontaneous abortion, and many other ailments (26, 27). We found that both rice and vegetables contained, on average, 30 μg/kg of cadmium. The maximum level of cadmium permitted by the Japanese Government and by the Codex Alimentarius in rice is 400 μg/kg (28, 29), and for leafy vegetables, the limit recommended by Codex Alimentarius is 200 μg/kg (29), which indicates that the concentration of cadmium in Bangladeshi foods is still within the safe limit. However, since rice is a staple food in Bangladesh and is consumed twice a day in large quantities (average consumption of adults is 1,500 g of cooked rice per day) (30), it could result in accumulation of cadmium in the body within a short period. Iron-deficient individuals have higher uptake of cadmium than those with balanced iron reserve (31). Thus, menstruating women and children with low iron stores have higher resorption of cadmium because of increased expression of DCT-1, a metal ion transporter (32). Half-life of cadmium is 10 years which accumulates in the body and is not metabolized and detoxified as arsenic (27). Thus, chronic exposure to cadmium in staple food, especially among rice-eating population, would have serious public-health consequences. In our study, both rice and vegetables contained cadmium. The presence of cadmium in rice and vegetables in Matlab could be due to the use of chemical fertilizers, e.g. triple superphosphate, and pesticides (composed of cadmium or its derivatives) for the cultivation of paddy and vegetables. Toxic metal deposition in urban and rural areas is a problem because of industrial wastage, sewage sludge, chemical fertilizers, and pesticides. As a consequence, grazing animals are exposed to pollutants deposited on pastures. Thus, humans are additionally exposed to cadmium-contaminated animal products, especially from meat and milk (33–35).

The mitigation of arsenic from the food-chain can be comprehended using different types of cooking methods and by changing the social behaviours of the communities through raising public awareness. Since the cooking practices do not affect concentration of cadmium in food, efforts should be focused on changing the types of fertilizers and pesticides used that may lower the concentration of cadmium in food, e.g. using rice variety that requires lower use of pesticides and fertilizers.

### Limitations

The limitations of the study are: (a) food samples (rice and vegetables) collected were not well-representative of all types of food consumed in other areas of Bangladesh and (b) fish or pulses were not included in the study that are also consumed in the daily diet.

### Conclusions

This exploratory study has shown that arsenic and cadmium have entered the food-chain of Bangladesh and identified rice and vegetables as major sources of arsenic and cadmium. Both arsenic and cadmium are easily available in the environment, and the exposure via food, water, and other occupational sources can contribute to a spectrum of diseases (36). Since exposure to toxic metals has become an increasingly-recognized cause of morbidity globally (26, 27, 37), it is necessary to identify the additional toxicants, e.g. lead, manganese, chromium, and cobalt, that may enter into the food-chain from agricultural and industrial activities.

## ACKNOWLEDGEMENTS

This study was funded by ICDDR,B. ICDDR,B gratefully acknowledges the following donors which provide unrestricted support to the Centre's research efforts: Australian Agency for International Development (AusAID), Government of the People's Republic of Bangladesh, Canadian International Development Agency (CIDA), Embassy of the Kingdom of the Netherlands (EKN), Swedish International Development Cooperation Agency (Sida), and Department for International Development, UK (DFID).

The authors thank Dr. G.B. Nair for his support and are very grateful to Dr. Rubhana Raqib for her constructive suggestion, critique, and ideas to prepare the manuscript at different stages.

The first two authors contributed equally to construct the manuscript, and the other authors contributed by planning, editing, and reviewing and by adding potential information to make it more precise.
